# Cholera outbreak in Balochistan amidst flash floods: an impending public health crisis

**DOI:** 10.1097/JS9.0000000000000150

**Published:** 2023-02-16

**Authors:** Shizra Jawed, Muhammad Bilal Islam, Hashir Ali Awan, Irfan Ullah, Muhammad Sohaib Asghar

**Affiliations:** a Department of Internal Medicine, Dow Medical College, Karachi, Pakistan; b Department of Internal Medicine, Dow University of Health Sciences, Karachi, Pakistan; c Kabir Medical College, Gandhara University, Peshawar, Pakistan

**Keywords:** cholera, climate change, floods, infectious diseases, public health

## Abstract

Despite being the largest province in Pakistan due to inadequate and underdeveloped infrastructure, Balochistan has been the worst-inflicted region with biblical floods. Following these disastrous flash floods, a sudden rise in cholera cases was seen in the affected province. To overcome this public health crisis, the authorities must put in place a system to ensure food safety, an adequate supply of clean drinking water, and the provision of proper sanitation facilities for the locals.

Highlights

- Despite being the largest province in Pakistan due to inadequate and underdeveloped infrastructure, Balochistan has been the worst-inflicted region with biblical floods.
- Following these disastrous flash floods, a sudden rise in cholera cases was seen in the affected province.
- To overcome this public health crisis, the authorities must put in place a system to ensure food safety, an adequate supply of clean drinking water, and the provision of proper sanitation facilities for the locals.

Despite being the largest province in Pakistan due to inadequate and underdeveloped infrastructure, Balochistan has been the worst-inflicted region with biblical floods that have hit the South-Asian country this year. Breaking a 30-year-old record, this season’s monsoon rain has become an unprecedented nightmare for Balochistan, bringing widespread devastation across various districts. According to the national database, the unfavorable effects of torrential rain in the province have resulted in ∼72,000 houses being destroyed in the province, with 360,000 lives being affected. Furthermore, 600 of the overall 800 schools ruined by the floods are present in Balochistan, with around 103 healthcare institutions being destroyed. Since July 2022, about 1663 people have lost their lives, and 33 million have been afflicted by floods across the country^1^.

In a study by Mora *et al*.,^2^ it was found that over 58% of infectious diseases have been made worse by climate disasters such as floods, earthquakes, and droughts. They greatly disrupt health infrastructure and worsen sanitation, taking an enormous toll on the public healthcare system of developing countries. The damage to existing medical facilities is expected to intensify healthcare challenges, especially when the drinking supply systems were significantly damaged in the floods, reducing access to safe and clean water across the province^1^.

Following these disastrous flash floods, a sudden rise in cholera cases was seen in the affected province. Cholera is an acute intestinal infection caused by the bacterium *Vibrio cholerae*, which can be found in contaminated water. Driven by a lack of access to safe drinking water and poor sanitation, it is a highly contagious illness that can produce severe acute watery diarrhea with significant morbidity and mortality, and it can spread quickly depending on the frequency of exposure^3^. It affects ∼2.9 million people annually, resulting in 95,000 deaths worldwide, mainly in low-income and middle-income countries, and has wreaked devastation on practically every continent for ages, with a higher frequency in underdeveloped nations due to poverty^4^. Pakistan, endemic to cholera, continues to be at significant risk for subsequent fatal cholera outbreaks, especially during the ongoing monsoon season, creating havoc and panic across different provinces. Heavy rain and flooding can increase exposure to vibrio cholera bacterium, which thrives in moist settings, and on average, a 10 mm increase in weekly accumulated precipitation leads to a rise of between 1.5 and 3.5% of recorded cholera cases, significant at 1% level^5^.

Today, the burden of cholera in Pakistan is high, and Balochistan seems to be a major locus for this disease burden. An unprecedented rise in cholera has been observed in 16 districts of the Balochistan province (southwest Pakistan), with more than 13,000 suspected cases reported across the province. The current report suggests 10 alarming deaths in the flood-affected district Zhob and Khuzdar within a week, making it a rising public health threat^6^. The WHO has rung alarm bells, and it is high time the healthcare administration takes immediate steps to combat the centuries-old challenge of cholera.

The sheer number of infectious diseases and pathways for their transmission is made worse by climatic risks highlights the severity of the threat that climate change poses to human health and the urgent need for decisive action^2^. Various NGO’s across the country have stood up to embark on the journey to help and provide aid to the flood victims, including gaining nationwide funds and international aid intervention. Pakistan Army, civil administration, and the Frontier Constabulary have been vigorously organizing rescue operations while waterboats and helicopters are also being used in this mission^1^.

To overcome this public health crisis, the authorities must put in place a system to ensure food safety, an adequate supply of clean drinking water, and the provision of proper sanitation facilities for the locals. Additionally, the widespread use of oral cholera vaccines can significantly aid in reducing infections during an escalating outbreak. To curb the spread of disease in flood-affected areas, around 6000 vaccines have been provided by UNICEF in the Lasbela district^7^. Public awareness on the importance of seeking medical care, oral rehydration use, and individual hygiene is imperative to keep the transmission rate to a minimum. Lastly, greater concentration on establishing stringent disease surveillance policies and increasing the reporting system will make it easier to devise an appropriate plan to control its spread.

## Ethical approval

Not applicable.

## Sources of funding

None.

## Authors’ contribution

S.J. and M.B.I.: conceived the idea; H.A.A., M.S.A., I.U., and M.B.I.: collected the data; S.B.I. and M.S.A.: reviewed literature; I.U. and S.J.: write-up of the manuscript; M.S.A.: reviewed and revised the manuscript for intellectual content critically. All authors approved the final version of the manuscript.

## Conflicts of interest disclosure

There are no conflicts of interest.

## Guarantor

Muhammad Sohaib Asghar.

## Data availability

No data has been used to demonstrate this study’s findings.
